# Olfactory disorders in COVID-19 patients as a prognostic factor: a systematic review

**DOI:** 10.1186/s43163-022-00360-5

**Published:** 2023-01-23

**Authors:** Farnaz Hashemian, Mona Rezazadeh, Amin Doosti Irani, Leila Moradi

**Affiliations:** 1 Otolaryngology–Head and Neck Surgery Department, School of Medicine, Hamadan University of Medical Sciences, Hamadan, Iran; 2 Department of Epidemiology, School of Public Health, Hamadan University of Medical Sciences, Hamadan, Iran

**Keywords:** Coronavirus, Olfaction disorders, Taste disorders, Prognosis, Systematic review, Meta-analysis

## Abstract

**Background:**

The reduction, loss, or impaired sense of smell and taste is common in patients with COVID-19. We aimed to investigate olfactory disorders (ODs) in patients with COVID-19 as a prognostic factor.

**Methods:**

In this systematic review and meta-analysis, studies that assessed ODs in patients with COVID-19 were included. International databases, including PubMed, Embase, MEDLINE, Web of Science, and Scopus, were searched up to 20 March 2021. The random-effects model was used to combine the results of studies. Results were reported with a 95% confidence interval.

**Results:**

In this study, out of 724 references, ten studies had the inclusion criteria. The odds of death in patients with the OD were 69% lower than in those without the ODs (*OR* = 0.31, 95% *CI*: 0.14, 0.69), and OD increased the odds of positive polymerase chain reaction (PCR) test (*OR* = 13.34, 95% *CI*: 4.2, 42.37).

**Conclusions:**

The findings of our study showed that OD had an inverse and significant relationship with death in COVID-19 patients, and the patients with OD seemed to have a lower risk of mortality.

## Background

COVID-19 is an infectious disease with a high rate of transmission and spread, which causes increased morbidity and mortality in patients. According to the studies, COVID-19 disease has various clinical symptoms, including dry cough, with or without fever, chills, sore throat and fatigue, shortness of breath, hypoxemia, and gastrointestinal symptoms such as nausea, vomiting, and diarrhea [1]. The severe form of COVID-19 can lead to death due to significant alveolar damage and progressive respiratory failure [2–4]. Many patients, especially those with its severe form, have underlying diseases such as high blood pressure, cardiovascular disease, chronic lung disease, diabetes, obesity, immunodeficiency, and malignancies [1].

The reduction, loss, or impaired sense of smell and taste is common in patients with COVID-19 [5–8]. Anosmia and hyposmia, the inability or reduced ability to smell, and parosmia, or the sense of unpleasant smell, are a disorder that affects 3 to 20% of the population and increases with age [9]. These patients usually have difficulty detecting olfactory and gustatory functions [10], which are often ignored by medical professionals [11]. It is noteworthy that acute anosmia usually occurs following viral infections or trauma [12].

Upper respiratory tract infection is one of the common causes of smell loss, which includes 22 to 36% of cases [13]. It is believed that the mechanism of olfactory disorder (OD) in anosmia is due to damage in the olfactory epithelium by the virus and is independent of nasal congestion [9]. It has been observed that in patients with a reduced sense of smell after infection, the volume of olfactory bulbs, which is related to olfactory function, decrease and even acts as a prognostic factor in these patients [14]. In 2020, clinicians realized that many patients with COVID-19 had experienced a decrease in the sense of smell or taste [15], and its incidence was reported to be 22 to 68% [5, 6].

A review of the reduced sense of smell following infection suggests the hypothesis that the virus may cause an inflammatory reaction in the nasal mucosa or direct damage to the olfactory neuroepithelium. However, the loss of the sense of smell in patients with COVID-19 can be significant without the presence of other rhinological symptoms or signs of inflammation. Finally, the researchers concluded that the pathological mechanism is still unknown. Still, it is most likely that the primary infection of nonolfactory nerve epithelial cells causes damage to the olfactory nerve. Decreased sense of smell and taste can be good signs and symptoms for early diagnosis of COVID-19 and the self-quarantine process. The prognosis of reduced sense of smell and taste in COVID-19 is better than in other viral infections [8]. It has been also noted that the use of sodium gluconate may improve the OD post-COVID-19 infection [16]. Considering the importance of a comprehensive investigation of COVID-19, this systematic review and meta-analysis aimed to assess olfactory and taste disorders in COVID-19 as a prognostic factor.

## Methods

This systematic review and meta-analysis was conducted and reported based on the PRISMA statement [17]. This study was approved by the ethics committee of Hamadan University of Medical Sciences (IR.UMSHA.REC.1399.1010).

### Eligibility criteria

All cross-sectional, case-control, and cohort studies that investigated the prognostic factors of COVID-19 disease were included in the study, regardless of the date, place, and language of publication.

### Search strategy

In this study, international electronic databases including PubMed, Embase, MEDLINE, Web of Science, and Scopus were searched using a pre-designed search strategy. The following keywords were used in the search strategy: COVID-19, SARS-CoV-2, olfaction disorders, dysgeusia, mortality, intubation, intensive care unit (ICU), critical care, critical care nursing, ICU requirements, and duration of hospitalization. To access more resources, we explored the World Health Organization (WHO), the Centers for Disease Control and Prevention (CDC), the European Centre for Disease Prevention and Control (ECDC), and the website of conferences related to COVID-19. The list of sources of selected articles was reviewed, and we contacted the authors of the included studies to access the unpublished articles.

### Selection of studies

Two researchers (MR and LM) were independently responsible for screening the references based on the title and abstract. Any disagreement between the researchers was resolved through negotiation or with the supervisor’s guidance.

### Data extraction

After selecting the studies, the required variables such as the name of the first author, place and time of the study, type of study, sample size, demographic characteristics, and the number of patients with COVID-19 who suffered the consequences of intubation and hospitalization in the ICU were entered in an electronic checklist.

### Risk of bias assessment

In this study, the Newcastle Ottawa scale (NOS) [18] was used to evaluate possible biases depending on the type of study. By this scale, in case-control/cohort studies, three domains including selection, comparability, and exposure/outcome were evaluated. In cross-sectional studies, a modified version of this scale was used, and three domains including selection, comparability, and outcome were evaluated. In this scale, each item of the mentioned domains with high quality was given a star. Overall studies with seven and more stars were low, and studies with six and lower stars were high risk of bias. The evaluation was done by two researchers (MR and LM) independently. Any disagreement between the researchers was resolved through negotiation or with the supervisor’s guidance.

### The outcomes

The outcomes investigated in this study were olfactory and taste disorders in patients with COVID-19. In case-control and cross-sectional studies, the odds ratio (OR) with a 95% confidence interval of the relationship between smell or taste disorder and the prognosis of patients was extracted. In cohort studies, the relative risk (RR) or odds ratio was used to assess the relationship between olfactory and taste disorders and disease prognosis.

Also, the mean and standard deviation of the hospitalization period in patients with olfactory or taste disorders were extracted. The statistical heterogeneity among the results of the included studies was tested using the chi-square test and the quantities by *I*^2^ statistics [19].

### Data analysis

Review Manager 5 and Stata 14.2 (StataCorp, TX, USA) were used for data analysis. In this study, the random-effects model was used to report the results of the meta-analysis. The results were reported with a 95% confidence interval (CI).

## Results

**Fig. 1:**
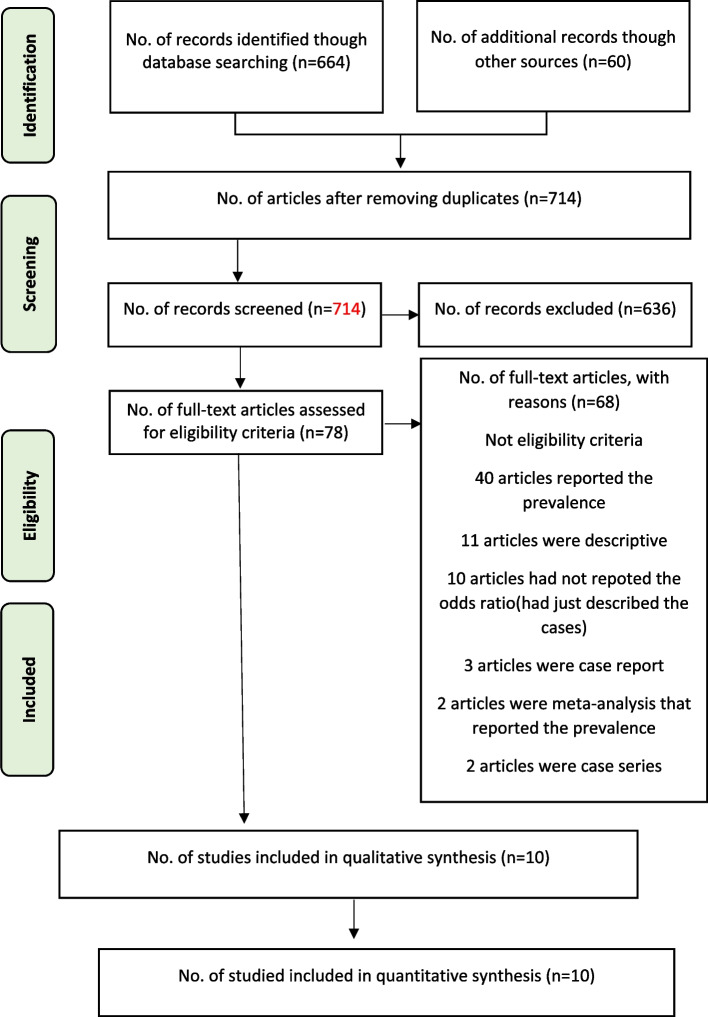
PRISMA flowchart

In this systematic review, out of 724 references, ten studies finally had the inclusion criteria [20–29] (Fig. 1). The total number of participants in the included studies was 1035. Both sexes were included in the studies, and the mean age was from 39 to 67 years. As shown in Table 1, 1599 individuals in these ten studies had ODs, 1403 had gustatory disorders, and 1261 had both disorders.

**Table 1:** The characteristics of included studies

Authors	Type of study	Country	Study period	Sample size	Sex	Age	In or outpatients	Anosmia	Taste loss	Both disorders	Death—anosmia	Death—taste loss	ICU—taste loss	ICU—anosmia	Intubation—anosmia	Intubation—taste loss	NOS—scale	Quality
Amanat (2021) [20]	Retrospective cohort	Iran	112	873	Both	60.71	Hospitalized	232	561	561	65						********	High
Avcı (2020) [21]		Turkey	40	1534	Both	39.44	Both	529	300								********	High
Etessam (2021)		Spain		5868	Both	64.2	Both	377	404	505	1079			303			*******	High
Petrocelli (2020) [23]		Italy	18	300	Both	43.6	Both	141	20	164	0	0		27	0	0	********	High
Talavera (2020) [24]		Spain	35	576	Both	67.2	Hospitalized	146			5						********	High
Yan (a) (2020) [25]		USA	36	169	Both	53.3	Both	7				3			0		*********	High
Vaira (2020) [26, 30]		England	20	106	Both	49.6	Both	44			0	0					*******	High
Carignan (2020) [27]		Canada	14	268	Both	57.1	Both	69	68		0	0			0	0	*********	High
Yan (2020) [25]	Cross-sectional	USA	23	262	Both	45	Hospitalized	40			0	0	0	0	0		********	High
Corbellini (2020) [28]		Spain	10	79	Both	61.6	Both	14	50	31	0	0			5		********	High

**Fig. 2:**
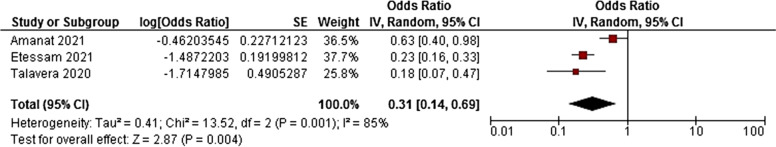
Forest plot for the association of OD with death in COVID-19

The presence of OD had a significant association with the reduced odds of death. The chance of death in patients with OD was 69% lower than in those without OD (*OR* = 0.31, 95% *CI*: 0.14, 0.69) (Fig. 2).

**Fig. 3:**
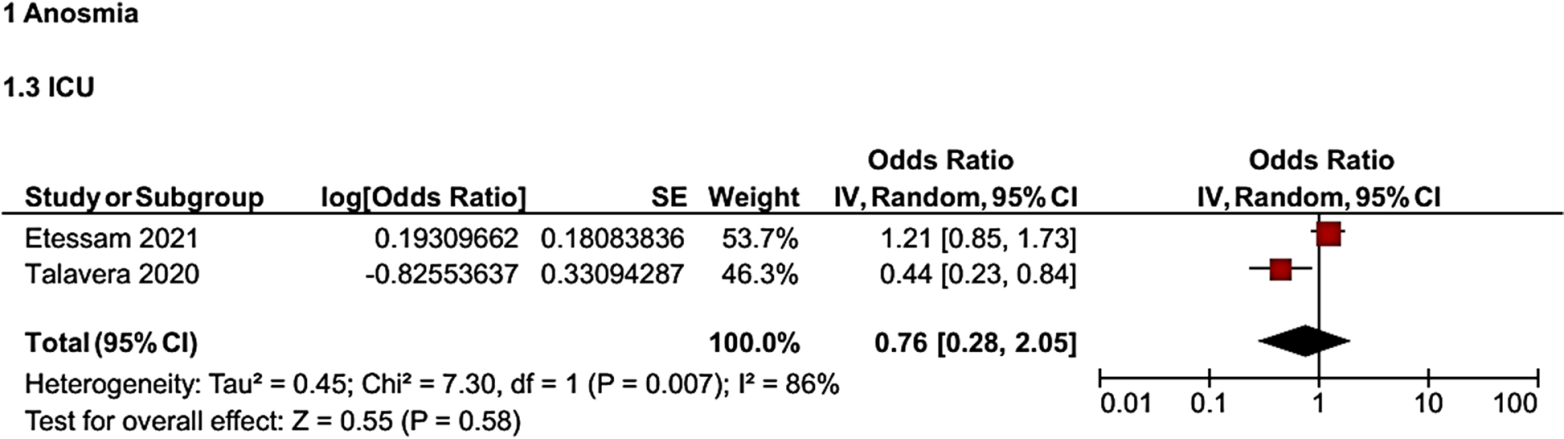
Forest plot for the association of OD with hospitalization in ICU in COVID-19

It was found that the odds of hospitalization in the ICU for patients with OD were 24% lower than for those without OD (*OR* = 0.76, 95% *CI*: 0.28, 2.05). However, this association was not statistically significant (Fig. 3).

**Fig. 4:**
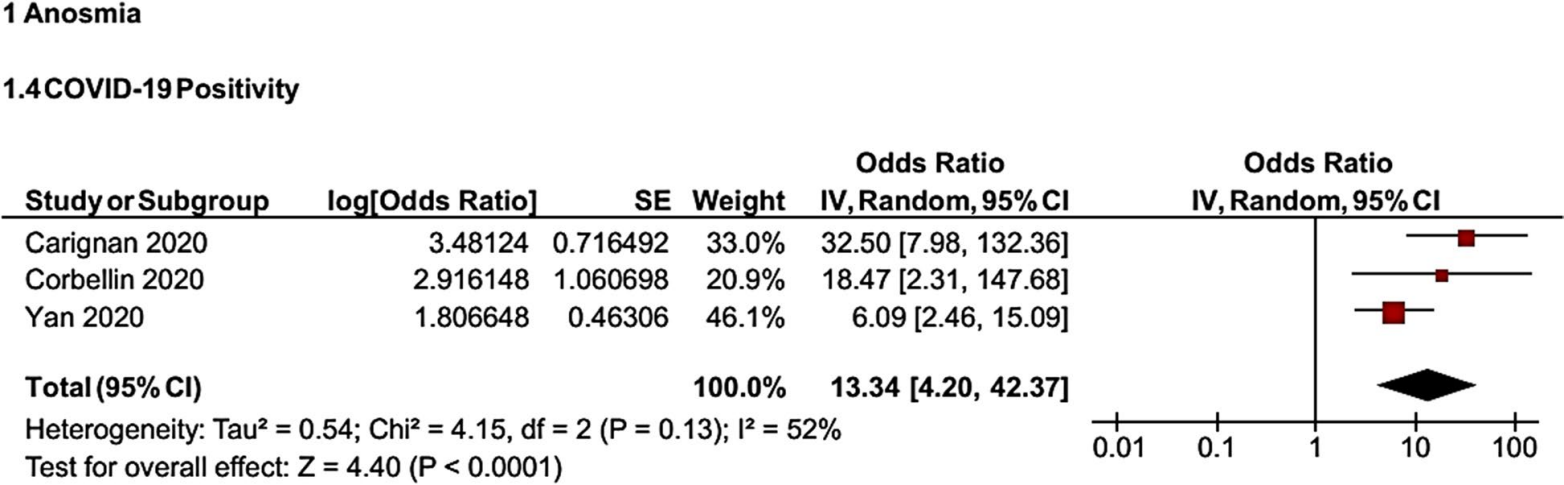
Forest plot for the association of OD with PCR test positivity in COVID-19

Assessing the relationship between OD and PCR test positivity in COVID-19 patients showed that there is a significant association between OD and PCR test positivity (*OR* = 13.34, 95% *CI*: 4.2, 42.37) (Fig. 4).

**Fig. 5:**
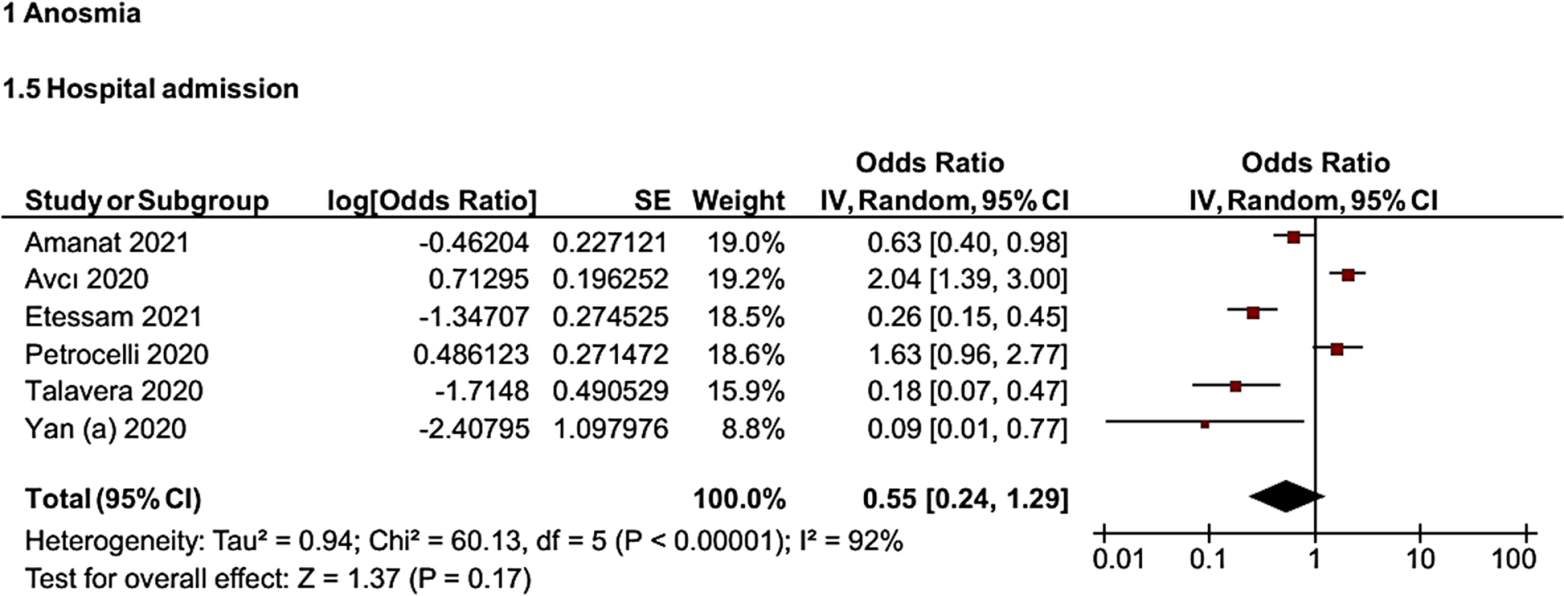
Forest plot for the association of OD with hospital admission in COVID-19

It was found that the odds of hospital admission for patients with OD were 45% lower than that of patients without OD (*OR* = 0.55, 95% *CI*: 0.24–1.29). However, this relationship was not statistically significant (Fig. 5).

## Discussion

The present study aimed to investigate OD in COVID-19 as a prognostic factor. In examining the relationship between OD and death in COVID-19 patients, it was found that these two variables had an inverse and significant relationship, and the patients with OD seemed to have a lower risk of mortality. Also, there was a direct and significant relationship between OD and PCR test positivity, and OD increased the risk of PCR test positivity. However, no significant association was noted between ODs with ICU hospitalization and hospital admission.

Anosmia has been described as one of the characteristic symptoms of COVID-19. It is even considered a key marker for the diagnosis of COVID-19 according to the CDC [31]. Despite having an estimated frequency of 52.7% [32] and being a clinical marker of COVID-19, there is a shortage of evidence on its relationship with the prognosis of COVID-19 [25]. The previous study associated anosmia with a mild course of COVID-19 and described an association between anosmia and an inverse probability of hospitalization [25]. In contrast, other studies have shown no significant relationship between the presence of olfactory changes and the severity of COVID-19. Indeed, some even suggested that the persistence of severe olfactory dysfunction could be associated with hospitalization after 20 days. Notably, these studies did not assess other known risk factors with the potential contribution to the outcomes [25, 30, 33].

The symptom of anosmia itself usually appears early in the infection [34–36], with a mean of 4.4 days after clinical onset and a mean duration of approximately 9 days, as described in a previous French study [25]. Anosmia is present in 60.9% of patients from the first day. In some cases, it has been introduced as the only symptom of the disease [35, 36]. That may be due to public health concerns, because patients with anosmia may be unaware of the condition and have a significant role in the spread of infection [37, 38].

Previous studies, in line of our results, showed that patients with anosmia had lower mortality [32, 34, 39–41]. These features have been independently associated with a better prognosis for COVID-19 [42–49]. Although the underlying causes of this association remain unknown, this finding could be related to different clinical manifestations.

In the previous studies, anosmia was independently associated with a higher probability of having a cough and myalgia [24, 50]. Although the pathophysiology of these symptoms is unknown, a greater frequency of systemic symptoms may be associated with an increased systemic response or viral replication and, therefore, a sign of an efficient innate immune response and a better prognosis [39, 48]. In another study, it is essential to emphasize that rhinorrhea, a common cause that can alter smell, was not significantly associated with anosmia [34]. Although anosmia has been associated with rhinorrhea and dysgeusia [25, 48] and myalgia [30], some experts believe that dysgeusia may be caused by an OD rather than affecting taste [51].

In different studies, it has also been stated that patients with anosmia had higher regulated levels of lymphocytes and hemoglobin, and lower levels of d-dimer at admission [24, 52]. The results also showed a higher hospitalization period, lymphocytes, hemoglobin, and glomerular filtration rate levels and lower d-dimer and C-reactive protein (CRP). Further changes in these parameters have been associated with a worse prognosis and an indirect measure of the systemic inflammatory response [15, 41–46, 52]. Some authors recommended monitoring these parameters in managing COVID-19 patients [53].

Similarly, patients with anosmia were admitted to the ICU less often, but there was no significant difference in our study [54]. This finding was consistent with the results of other authors who described a milder disease course in patients with anosmia [7, 25, 53]. We encourage investigators to assess whether the clinical presentation of outpatients with anosmia was also different from those without it.

While the pathophysiological mechanism of OD caused by COVID-19 is still not fully understood, a critical step is probably the interaction between the COVID-19 protein and the angiotensin-converting enzyme 2 (ACE2) receptor. The intense expression is in the olfactory neuron epithelium [55–57]. ACE2 expression is significantly absent in olfactory sensory neurons, suggesting an indirect effect of SARS-CoV-2 infection on olfactory function, possibly due to loss of integrity in the neuroepithelium, or ionic imbalance interferes with olfactory signals [55–57]. Regarding the association between OD and morbidity/mortality, existing studies have provided inconsistent, although significant findings indicate that patients with OD had a more benign immune response profile to COVID-19 infection. Specifically, patients with OD can have less lymphopenia, higher albumin and hemoglobin, and lower D-dimer and C-RP. They have local nonsystemic inflammatory reactions in the upper airways, as shown by radiography [7, 25].

Although taste disturbance may be a biomarker for the prognosis of COVID-19, OD was prioritized for this study because, as reported in studies, taste loss is likely a secondary condition to OD [33, 58]. In addition, we found 1599 patients with olfactory, 1403 with gustatory, and 1261 with both disorders. Eventually, due to data limitations, no significant results were obtained. For this reason, it seems that the existence of more studies that lead to the examination of these variables in a larger sample size will be more helpful in the final conclusion.

Limitations of this study included the differences in studies’ quality, targeted focus on smell reduction as a unique predictor, lack of standardized OD reporting protocols and definitions globally, heterogeneity of patient populations, different treatment protocols, and hospitalizations. Depending on geographic location and evolving treatment regimens (e.g., delaying intubation even as hypoxia levels increase), OD as a recognized symptom of COVID-19 prompts early assessment and intervention and improves outcomes. There are also many asymptomatic and undiagnosed patients with COVID-19, and unknown asymptomatic outbreaks are a problematic factor. Age is another critical confounder, given that older age is associated with lower rates of OD [59, 60] and worse disease outcomes [1, 61–64]. Although we did not have access to precise data to include age in our analysis, two previous studies [24, 62] (898 patients in total) showed through multivariate analyses that OD was a predictor of better outcomes even after adjusting for age. Further research must clarify whether the association between OD and COVID-19 outcomes is age dependent.

## Conclusions

The findings of our study showed that OD had an inverse and significant relationship with death in COVID-19 patients, and the patients with OD seemed to have a lower risk of mortality. It was also found that OD increased the risk of PCR test positivity.

## Data Availability

The datasets used and/or analyzed during the present study are available from the corresponding author upon reasonable request.
